# Enduring effect of abuse: Childhood maltreatment links to altered theory of mind network among adults

**DOI:** 10.1002/hbm.25787

**Published:** 2022-01-28

**Authors:** Yajing Pang, Shanshan Zhao, Zhihui Li, Nan Li, Jiarui Yu, Rui Zhang, Fengmei Lu, Heng Chen, Fengchun Wu, Wei Zheng, Jingjing Gao, Yongfeng Yang, Huawang Wu, Jiaojian Wang

**Affiliations:** ^1^ School of Electrical Engineering Zhengzhou University Zhengzhou China; ^2^ The Clinical Hospital of Chengdu Brain Science Institute, School of Life Science and Technology University of Electronic Science and Technology of China Chengdu China; ^3^ School of medicine Guizhou University Guiyang China; ^4^ The Affiliated Brain Hospital of Guangzhou Medical University (Guangzhou Huiai Hospital) Guangzhou China; ^5^ Guangdong Engineering Technology Research Center for Translational Medicine of Mental Disorders Guangzhou China; ^6^ School of Information and Communication Engineer University of Electronic Science and Technology of China Chengdu China; ^7^ Department of Psychiatry, Henan Mental Hospital The Second Affiliated Hospital of Xinxiang Medical University Xinxiang China; ^8^ Henan Key Lab of Biological Psychiatry Xinxiang Medical University Xinxiang China; ^9^ International Joint Research Laboratory for Psychiatry and Neuroscience of Henan Xinxiang China; ^10^ State Key Laboratory of Primate Biomedical Research, Institute of Primate Translational Medicine Kunming University of Science and Technology Kunming China

**Keywords:** childhood maltreatment, functional connectivity, impulsivity, theory of mind

## Abstract

Childhood maltreatment (CM) confers a great risk of maladaptive development outcomes later in life, however, the neurobiological mechanism underlying this vulnerability is still unclear. The present study aimed to investigate the long‐term consequences of CM on neural connectivity while controlling for psychiatric conditions, medication, and, substance abuse. A sample including adults with (*n* = 40) and without CM (*n* = 50) completed Childhood Trauma Questionnaire (CTQ), personality questionnaires, and resting‐state functional magnetic resonance imaging scan were recruited for the current study. The whole‐brain functional connectivity (FC) was evaluated using an unbiased, data‐driven, multivariate pattern analysis method. Relative to controls, adults with CM suffered a higher level of temperament and impulsivity and showed decreased FC between the insula and superior temporal gyrus (STG) and between inferior parietal lobule (IPL) and middle frontal gyrus, STG, and dorsal anterior cingulate cortex (dACC), while increased FC between IPL and cuneus and superior frontal gyrus (SFG) regions. The FCs of IPL with dACC and SFG were correlated with the anxious and cyclothymic temperament and attentional impulsivity. Moreover, these FCs partially mediated the relationship between CM and attentional impulsivity. Our results suggest that CM has a significant effect on the modulation of FC within theory of mind (ToM) network even decades later in adulthood, and inform a new framework to account for how CM results in the development of impulsivity. The novel findings reveal the neurobiological consequences of CM and provide new clues to the prevention and intervention strategy to reduce the risk of the development of psychopathology.

## Graphical abstract

1. CM has a significant effect on the modulation of functional connectivity within theory of mind (ToM) network even decades later in adulthood.

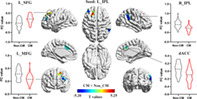

2. Cognitive ToM connectomes mediate the relationship between CM and attentional impulsivity.

## 1 INTRODUCTION

Childhood maltreatment (CM) is a common, detrimental public health concern (Gilbert et al., 2009). It is well established that CM confers vulnerability to maladaptive developmental outcomes (Morgan & Gayer‐Anderson, 2016), such as deficits in cognitive, emotional, social, and behavioral functioning (Bick & Nelson, 2016), maladaptive personality traits (Fletcher & Schurer, 2017), and the vulnerability to development of psychopathology across a lifetime (Nusslock & Miller, 2016). However, through what mechanism does early adverse experiences so strongly impact development in later life has not been clearly examined. A key breakthrough discovery is that CM changes trajectories of brain development (Teicher & Samson, 2013). Brain injury in children due to maltreatment, from the slight to the glaringly clear, has been well documented from early childhood, and can continue to influence development into the third decade of life (Chong, Ng, Lee, & Zhou, 2017). Therefore, characterizing the long‐term neurobiological consequence of CM is urgently needed for developing better prevention strategies and targeted treatment to reverse the CM‐induced brain change (Teicher, Samson, Anderson, & Ohashi, 2016).

There is growing evidence that CM is associated with structural and functional changes in the human brain, particularly in the regions subserving social cognition and emotion, including prefrontal cortex (PFC; Teicher et al., 2016), hippocampus (Dahmen et al., 2018), amygdala, insula, and anterior cingulate cortex (ACC) (Dannlowski et al., 2012; Lim, Radua, & Rubia, 2014). However, the brain is a functionally interconnected network (Fornito, Bullmore, & Zalesky, 2017), thus, examining the CM‐related neural connectome can advance our understanding of the neurodevelopmental consequences of CM. Recent functional connectivity (FC) studies have reported that CM was associated with increased amygdala FC with the hippocampus and PFC regions during an emotion‐matching task in adults (Jedd et al., 2015), and reduced FC between ventromedial PFC and insula during fear facial stimuli in adolescents (Hart et al., 2018). Resting‐state FC studies identified the CM‐induced increased connectivity in salience network (SN), reduced connectivity within the default mode network (DMN), and between DMN and SN in both adolescents and adults (Goetschius et al., 2020; Marusak, Etkin, & Thomason, 2015; Philip, Sweet, et al., 2013). Moreover, decreased FC between medial PFC and amygdala and increased large‐scale network modularity were associated with emotional abuse in girls (Cisler, 2017). The increased FC within theory of mind (ToM) network was influenced by the types and severity of abuse in women adults with CM (Boccadoro et al., 2019). The significant effect on a large of brain networks of CM was understandable due to the diverse influence in the social cognition–emotion processing of CM.

However, the abovementioned studies might miss critical neural signatures that existed outside such canonical networks as they focused on either priori defined seed regions or specific networks. Accordingly, an unbiased whole‐brain connectivity may be a more effective way to holistically elucidate the impact of maltreatment experiences on the brain. In addition, as the neural alterations following CM are mostly in line with the findings described in psychiatric disorders, controlling the typically co‐occurring psychiatric conditions is thus needed to determine the observed effects are a result of the maltreatment other than the associated psychiatric conditions or a combination or interaction of both (Hart & Rubia, 2012). Meanwhile, this can also provide new clues of latent vulnerability to the development of psychiatric disorders.

The present study aimed to use multivariate pattern analysis (MVPA), an unbiased whole‐brain data‐driven approach, to investigate the FC differences between healthy adults with and without a history of CM through resting‐state functional magnetic resonance imaging (rs‐fMRI) dataset. The utility of MVPA‐informed FC analysis has already been demonstrated in various clinical contexts such as autism spectrum disorder (Anteraper et al., 2019) and major depressive disorder (Wang et al., 2019). Based on the aforementioned literatures, we hypothesized that adults with CM experience would show altered FC implicated in social cognition and emotion. Moreover, given the effect of CM on the formation of personality (Fletcher & Schurer, 2017), and the neural circuit basis underlying individual differences in personality (Pang et al., 2019), mediation analysis was employed to explore the associations between CM, FC measures, and personality profiles.

## 2 METHODS AND MATERIALS

### 2.1 Participants

A total of 90 right‐handed adults aged 18 to 30 years, including 40 adults with CM experiences (CM group) and 50 age‐, gender‐, educational level‐matched comparison adults without CM exposures (non_CM group) were included in this study. The inclusion criteria of the participants were (a) a history of chronic trauma exposures (abuse and/or neglect) (CM group) or absence of such history (non‐CM group) before the age of 16; (b) free from any lifetime history of Axis I psychiatric disorder confirmed using the DSM‐V Structured Clinical Interview by two experienced psychiatrists; (c) without a family history of psychiatric disorders in any two lines of first‐ to third‐degree biological relatives. Exclusion criteria were (a) a history of neurological disorders or trauma; (b) substance or alcohol abuse or dependence; (c) current use of psychotropic medications; (d) significant medical illness; (e) MRI contraindications; and (f) inability to keep still during MRI scanning. This study was approved by the local medical ethics committee of the Affiliated Brain Hospital of Guangzhou Medical University. All participants provided written informed consent before any study procedure was initiated.

### 2.2 Child abuse

CM experience was assessed using a 28‐item self‐reported retrospective Childhood Trauma Questionnaire (CTQ; Bernstein et al., 2003). The CTQ includes five sub‐scales that evaluate five aspects of trauma exposures: emotional abuse, emotional neglect, sexual abuse, physical abuse, and physical neglect. A 5‐point Likert‐type scale is used for responses ranging from 1 (never true) to 5 (very often true). Participants were classified into the CM group when at least one subscale is above the threshold (emotional abuse ≥13, emotional neglect ≥15, sexual abuse ≥8, physical abuse ≥10, physical neglect ≥10; Bernstein et al., 1994). Reliability and validity of the CTQ have been previously demonstrated among Chinese (He, Zhong, Gao, Xiong, & Yao, 2019).

### 2.3 Personality assessment

#### 2.3.1 Temperament evaluation of Memphis, Pisa, Paris, and San Diego auto‐questionnaire

A short version of temperament evaluation of Memphis, Pisa, Paris, and San Diego auto‐questionnaire (TEMPS‐A) has been used to evaluate lifelong, milder aspects of bipolar symptomatology according to five temperaments: hyperthymic, dysthymic, cyclothymic, irritable, and anxious (Akiskal, Akiskal, Haykal, Manning, & Connor, 2005). Participants are required to respond with “true” or “false” to each of the 39 statements, with each dichotomous item scored 1 or 0. The higher the scores, the more obvious the temperament characteristics.

#### 2.3.2 Barratt Impulsiveness Scale

The personality construct of impulsivity was assessed using the Barratt Impulsiveness Scale‐11 (BIS‐11) (Patton, Stanford, & Barratt, 1995), a self‐rated questionnaire including 30 items, each rated on a 4‐point Likert‐type scale. Impulsivity is differentiated by three subscales: motor impulsiveness (tendency to act without thinking), nonplanning impulsivity (degree of focus on the immediate present), and attentional impulsiveness (inattention and cognitive instability; Arce & Santisteban, 2006). Higher scores indicate higher levels of impulsivity.

### 2.4 fMRI data acquisition

Resting‐state functional magnetic resonance imaging data were acquired on a 3 T Philips MRI scanner. All participants were instructed to relax with their eyes closed but stay awake, and to remain motionless. Foam pads and headphones were used to minimize head movement and scanner noise. Functional images were scanned using an echo‐planar imaging sequence with the following parameters: repetition time (TR) = 2,000 ms, echo time (TE) = 30 ms, slices = 33, matrix size = 64 × 64, flip angle = 90°, field of view = 220 × 220 mm^2^, thickness = 4 mm with 0.6 mm gap, and a total of 240 volumes.

### 2.5 Data preprocessing

Image preprocessing was performed using the DPARSF toolbox (http://rfmri.org/dpabi) based on SPM12 (http://www.fil.ion.ucl.ac.uk/spm/software/spm12/). The first 10 volumes were discarded before subsequent processing. The remaining 230 volumes were slice‐timing and head motion correction. All participants were retained under the head motion criteria of translation <2 mm or rotation <2° in any direction. The data were then spatially normalized to the standard Montreal Neurological Institute (MNI) space with a voxel size of 3 × 3 × 3 mm^3^. Nuisance covariates included white matter, cerebrospinal fluid, and the Friston‐24 parameters of head motion were regressed out from each voxel's time course. Afterwards, the images were spatial smoothed with full width at half maximum = 6 mm, and the linear detrend and filtering (0.01–0.08 Hz) were performed to reduce the influence of low‐frequency drift and high‐frequency physiological noise. In addition, the framewise displacement (FD) across time points for each subject was calculated to assess head motion. The mean FD values of CM group (0.10 ± 0.04) and non_CM group (0.10 ± 0.03) showed no difference (*t* = 0.55, *p* = .59). Finally, using the scrubbing method at an FD threshold of 0.5 mm, the “bad” time points and their 1‐back and 2‐forward neighbors were identified and corrected through cubic spline interpolation. The scrubbed time points of CM (0.60 ± 1.65) and non_CM group (1.57 ± 4.70) also showed no significant difference (*t* = 1.25, *p* = .22).

### 2.6 Multivoxel pattern analysis

Multivoxel pattern analysis (MVPA) is a whole‐brain connectomic method to conduct voxel‐wise resting‐state FC within the CONN toolbox. In detail, for each voxel, the FC was calculated between the time series within the given voxel and all other voxels in the brain. Principal component analysis was performed to reduce the high dimensionality of the data to 64 dimensions (i.e., spatial components) within each voxel. Thus, for each subject, each voxel of the MVPA‐derived map contained 64‐factor scores summarizing the voxel's FC with the rest of the brain. Subsequently, the first three of these 64 components were kept which explained the 97% of the between‐subjects variability in whole‐brain FC at each voxel. An *F*‐test was performed with each participant's three components at each voxel as dependent variables and with groups (i.e., CM and non‐CM) as independent variable, while age, gender, years of education, and mean FD were controlled. Multiple comparison correction was performed based on Gaussian random field (GRF) theory with cluster corrected *p* < .05 and a voxel height of *p* < .005.

Post hoc seed‐to‐voxel FC analysis was performed to explore what particular FC patterns differ between groups. The regions identified in the MVPA were defined as seed regions, with a sphere with radius = 6 mm was created. The Pearson's correlation coefficient was calculated between the time course of each seed region and the time course of all other voxels in the brain. Correlation coefficients were then converted into *z*‐scores using the Fisher *r*‐to‐*z* transformation to improve the Gaussianity of their distribution. Two sample *t*‐test was then conducted to examine the difference of FC between CM and non‐CM groups, while controlling age, gender, years of education, and mean FD. The result was multiple corrected using GRF correction with cluster corrected *p* < .05 and a voxel height of *p* < .005.

#### 2.6.1 Correlation analysis

Partial correlation analyses between the CM‐induced FC and personality indices (i.e., TEMPS and BIS scores) were performed in all subjects, with age, gender, and years of education as covariates. False discovery rate (FDR) correction *p* < .05 was implemented for multiple comparisons. In addition, the relationship between altered FC and subscales of CM was also examined (see Supporting Information for details).

#### 2.6.2 Mediation analysis

Inspired by the associations between CM, ToM connectome, and personality, we examined whether the FC of ToM network has a causal pathway from CM to maladaptive personality. Based on the results of correlation analysis, mediation analysis was thus performed with CM (i.e., CTQ total scores) as the independent variable (X), ToM connectomes (i.e., the FC of IPL‐dACC/SFG) as the mediator variable (M), and the personality profiles (i.e., anxious/cyclothymic temperament/attentional impulsivity scores) as the dependent variable (Y). Meanwhile, age, gender, and education years were treated as covariates. Mediation analysis was conducted using PROCESS macro implemented in SPSS software (SPSS Inc., Chicago, Illinois).

Following established methods for mediation (Rucker et al., 2011), the path analyses were conducted using a series of linear regression models (Figure 6a). In detail, the path analyses included the total effect of X on Y (path *c*), which consists of the direct effect of X on Y after controlling for M (path c′) and the indirect effect of X on Y through M [i.e., the product of path X→M (*a*) and M→Y (*b*); path a×b]. The mediation effect was evaluated whether path a×b is significantly different from zero, that is to say, whether a difference exists between the total effect (path c) and the direct effect (path c′) that accounts for M. Significance of the mediation effect was determined using a nonparametric bootstrapping method. The 95% confidence intervals (CIs) for medication effect were calculated via 5,000 bias‐corrected bootstraps (Preacher & Hayes, 2004). If the CI does not contain zero, then the mediation effect is considered to be significant (*p* < .05).

## 3 RESULTS

### 3.1 Demographic and behavioral variables

Demographic and behavioral characteristics of participants were presented in Table 1. CM and non_CM groups were matched with age, gender, and years of education. Significant differences between CM and non_CM groups were observed in CTQ, TEMPS, and BIS total and their subscale scores except hyperthymic temperament. Consistent with previous studies (Higashiyama et al., 2019; Liu, 2019), CM group showed higher levels of depressive, cyclothymic, irritable, and anxious temperament characteristics, and higher levels of attentional, motor, and nonplanning impulsivity than non_CM group. In maltreatment participants, the most common aspect of CM experience was physical neglect (62.5%), a proportion of 45% of traumatic participants experienced at least two forms of CM exposures. Moreover, the more types of CM exposures experienced, the higher level of temperament and impulsivity subjects had (Table S1).

**TABLE 1:** Demographic and behavioral characteristics

Variables	CM (*n* = 40)	Non‐CM (*n* = 50)	*p* value
Age (years)	22.90 ± 3.30	22.76 ± 3.46	.85^a^
Gender (male/female)	18/22	20/30	.63^b^
Education (years)	14.00 ± 2.40	14.00 ± 2.00	1^a^
*CTQ*			
Total score	44.70 ± 7.90	27.32 ± 1.82	<.0001^a^
Emotional abuse	8.43 ± 3.19	5.58 ± 0.84	<.0001^a^
Physical abuse	6.98 ± 2.36	5.14 ± 0.50	<.0001^a^
Sex abuse	5.9 ± 1.40	5.00 ± 0.00	<.0001^a^
Emotional neglect	13.55 ± 4.29	6.28 ± 1.28	<.0001^a^
Physical neglect	9.85 ± 2.60	5.32 ± 0.55	<.0001^a^
*CT exposures*, *n* (%)			
Emotional abuse	5 (12.5)		
Physical abuse	8 (20)		
Sex abuse	5 (12.5)		
Emotional neglect	19 (47.5)		
Physical neglect	25 (62.5)		
Single exposure	22 (55)		
Co‐types exposure	16 (40)		
Tri‐types exposures	2 (5)		
*TEMPS‐A*			
Total scores	38.35 ± 15.82	26.04 ± 9.97	<.0001^a^
Depressive temperament	8.78 ± 3.70	5.86 ± 2.26	<.0001^a^
Cyclothymic temperament	7.63 ± 5.05	3.60 ± 3.71	<.0001^a^
Hyperthymic temperament	10.78 ± 4.61	10.94 ± 3.54	.85^a^
Irritable temperament	4.28 ± 3.57	1.46 ± 1.96	<.0001^a^
Anxious temperament	6.90 ± 5.53	4.18 ± 3.52	.006^a^
*BIS*			
Total scores	65.35 ± 15.10	54.64 ± 14.12	.0008^a^
Attentional impulsivity	18.55 ± 3.71	16.04 ± 4.99	.01^a^
Motor impulsivity	22.73 ± 4.18	20.34 ± 4.61	.01^a^
Non‐planning impulsivity	27.63 ± 3.49	24.38 ± 4.76	.005^a^

*Note*: Values are mean ± *SD*.

Abbreviations: BIS, Barratt Impulsiveness Scale; CM, childhood maltreatment; CTQ, Childhood Trauma Questionnaire; TEMPS_A, Temperament Evaluation of Memphis, Pisa, Paris, and San Diego Auto‐questionnaire.

^a^ Two‐sample *t*‐test.

^b^ Chi‐square *t*‐test.

### 3.2 Altered FC associated with CM

**FIGURE 1:**
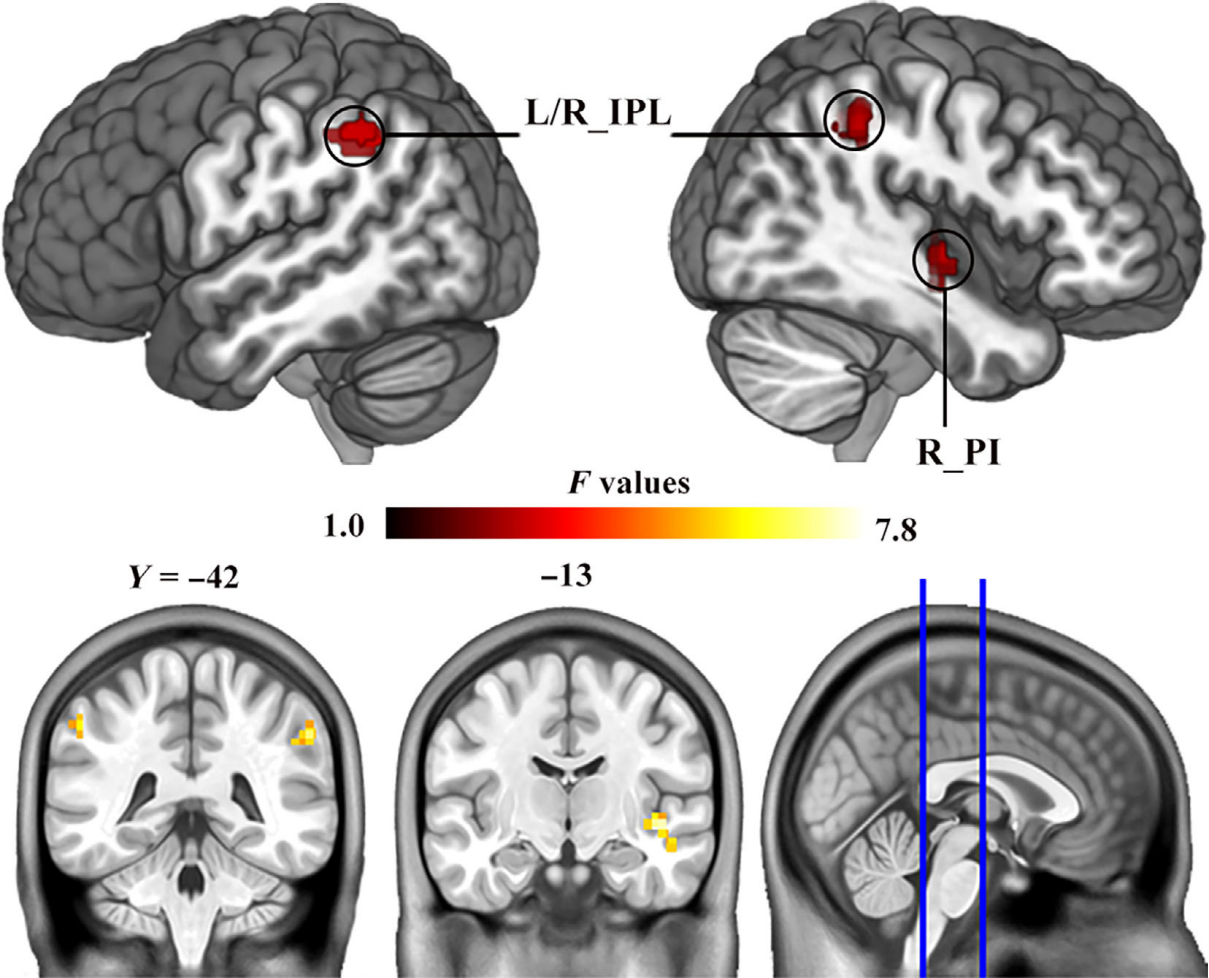
Results of MVPA. MVPA identified three brain regions that showing a significant difference in whole‐brain connectivity between CM and non_CM groups (voxel level *p* < .005, cluster level *p* < .05, GRF corrected). CM, childhood maltreatment; L, left; MVPA, multivoxel pattern analysis; IPL, inferior parietal lobule; PI, posterior insula; R, right

**FIGURE 2:**
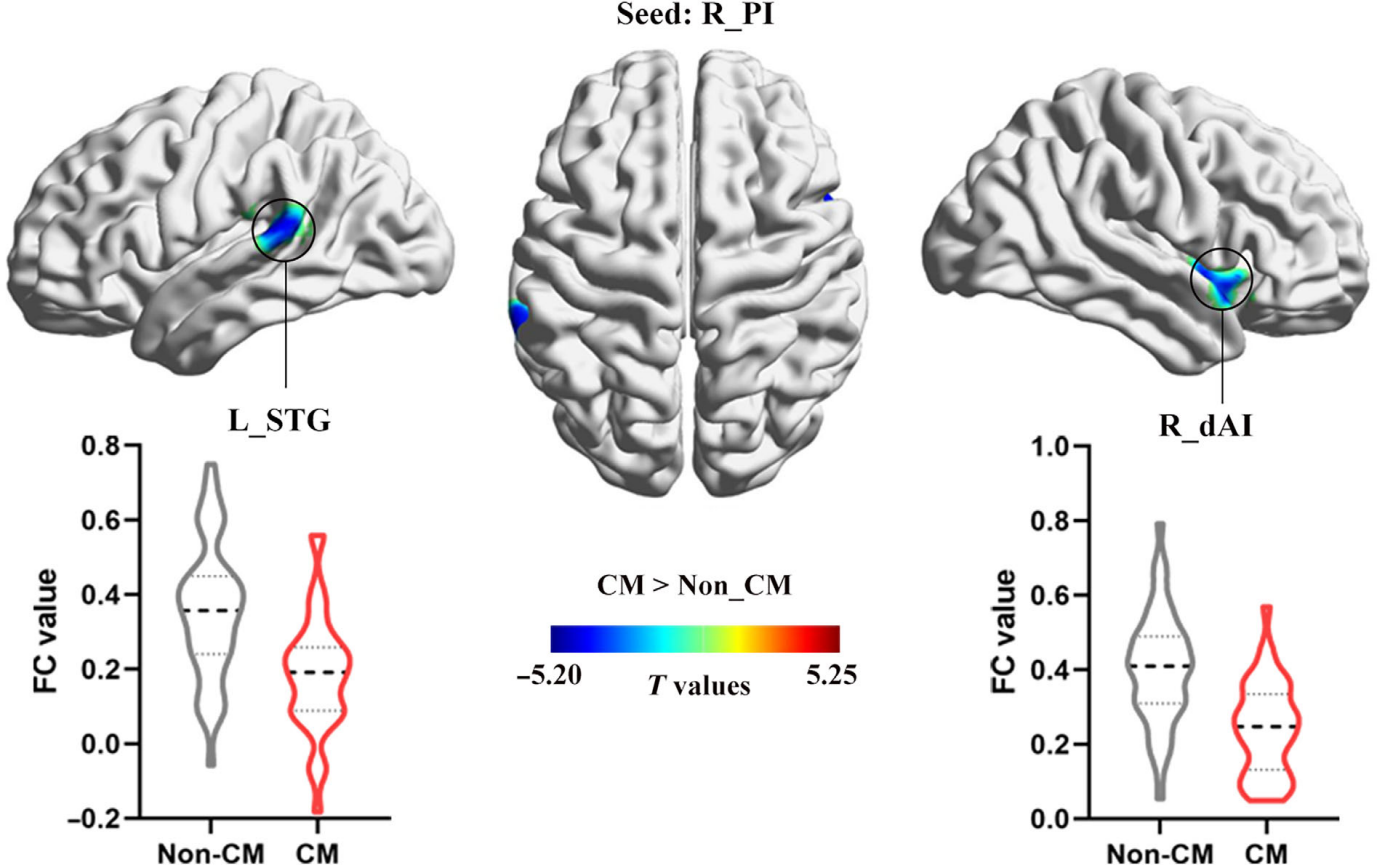
Group difference in FC of right PI between CM and non‐CM groups (voxel level *p* < .005, cluster level *p* < .05, GRF corrected). CM, childhood maltreatment; dAI, dorsal anterior insula; FC, functional connectivity; L, left; PI, posterior insula; R, right; STG, superior temporal gyrus

**FIGURE 3:**
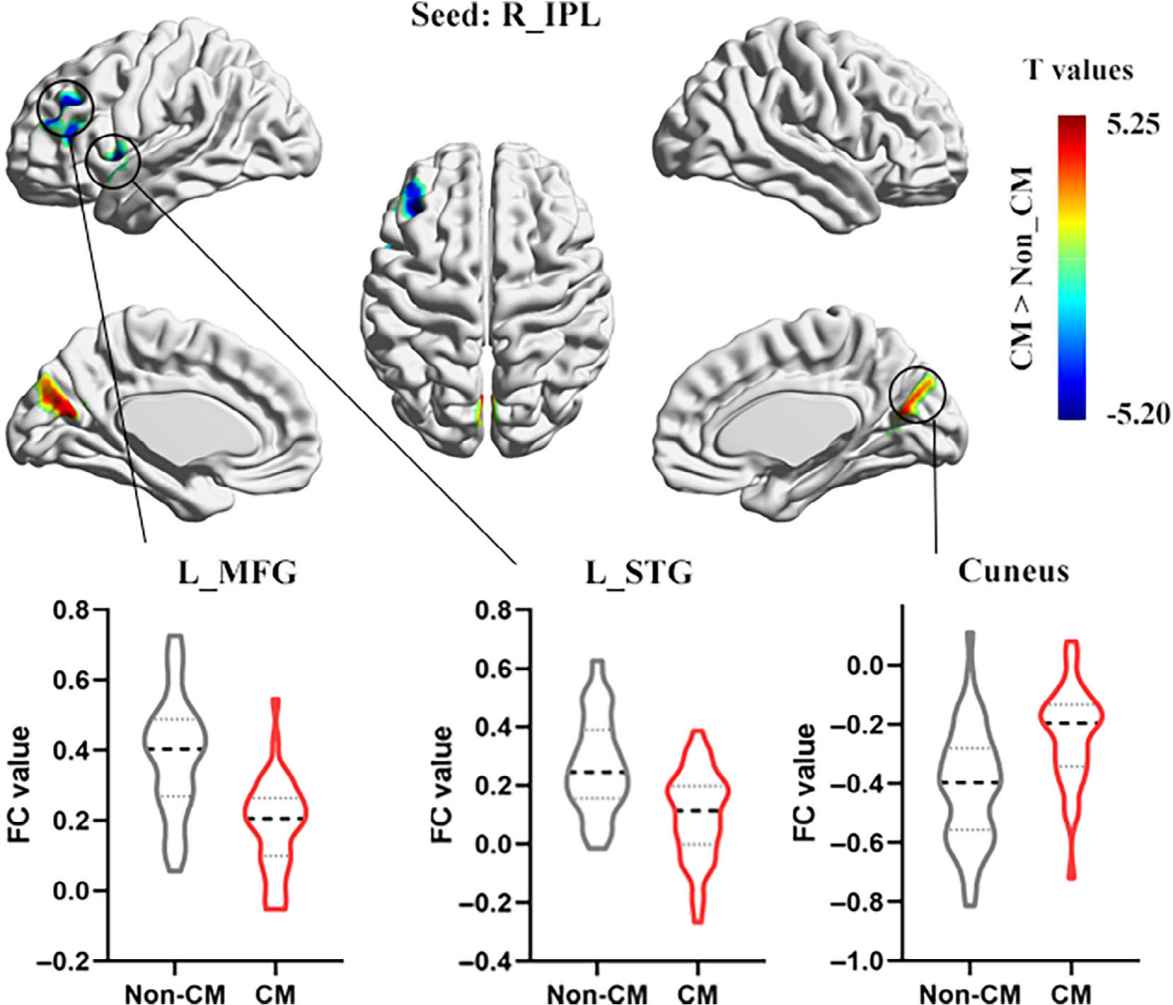
Group difference in FC of right IPL between CM and non‐CM groups (voxel level *p* < .005, cluster level *p* < .05, GRF corrected). CM, childhood maltreatment; FC, functional connectivity; IPL, inferior parietal lobule; L, left; MFG, middle frontal gyrus; R, right; STG, superior temporal gyrus

**FIGURE 4:**
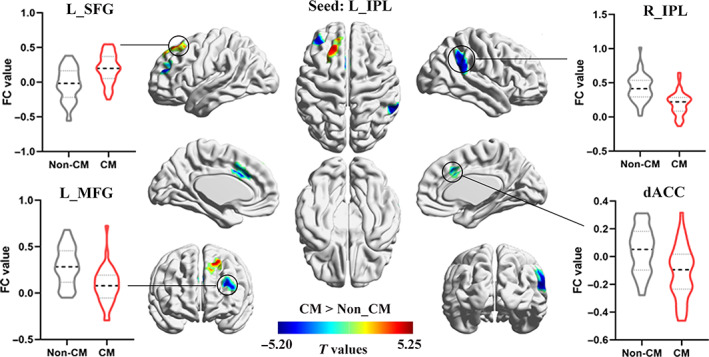
Group difference in FC of left IPL between CM and non‐CM groups (voxel level *p* < .005, cluster level *p* < .05, GRF corrected). CM, childhood maltreatment; dACC, dorsal anterior cingulate cortex; FC, functional connectivity; IPL, inferior parietal lobule; L, left; MFG, middle frontal gyrus; R, right; SFG, superior frontal gyrus

Whole‐brain MVPA results showed right posterior insula (PI) and bilateral inferior parietal lobule (IPL) with a significant CM effect (Figure 1, Table 2). For the right PI seed region, CM group showed decreased FC with right dorsal anterior insula (dAI) and left superior temporal gyrus (STG; Figure 2, Table 3). For the right IPL seed region, CM group showed increased FC with cuneus, and decreased FC with left STG and left middle frontal gyrus (MFG; Figure 3, Table 3). For the left IPL seed region, CM group showed increased FC with left superior frontal gyrus (SFG), and decreased FC with right IPL, left MFG, and dorsal ACC (dACC; Figure 4, Table 3). Furthermore, these FC results were not influenced by the kind of CM exposures (see Supporting Information for details, Figure S1). Last, the CM type specifically correlated FC was examined and found that the FC between the PI and dAI was positively correlated with physical neglect, while the FC between the IPL and cuneus was negatively correlated with emotional neglect (Figure S2).

**TABLE 2:** Brain regions identified by MVPA

Brain regions		Peak MNI coordinates			Cluster size (voxels)	*F* value
	Brodmann area	*x*	*y*	*z*		
R_PI	13	42	−12	−6	89	7.30
R_IPL	40	58	−40	38	93	7.30
L_IPL	40	−54	−48	44	98	6.48

Abbreviations: IPL, inferior parietal lobule; L, left; MVPA, multivoxel pattern analysis; PI, posterior insula; R, right.

**TABLE 3:** Changed FC with the seed regions identified by MVPA in CM group

Brain regions		Peak MNI coordinates			Cluster size (voxels)	*T* value
	Brodmann area	*x*	*y*	*z*		
*Seed: R_PI*						
R_dAI	22	48	9	−6	91	−4.42
L_STG	40	−66	−30	18	103	−4.20
*Seed: R_IPL*						
L_STG	22	−42	−15	−3	128	−4.16
Cuneus	31	−3	−69	18	116	4.45
L_MFG	46	−36	30	27	159	−5.61
*Seed: L_IPL*						
R_IPL	40	57	−45	39	229	−5.20
L_MFG	10	−33	39	24	124	−4.82
dACC	32	−12	30	30	91	−3.98
L_SFG	8	−21	36	54	115	5.25

Abbreviations: CM, childhood maltreatment; dACC, dorsal anterior cingulate cortex; dAI, dorsal anterior insula; IPL, inferior parietal lobule; L, left; MFG, middle frontal gyrus; MVPA, multivoxel pattern analysis; PI, posterior insula; R, right; SFG, superior frontal gyrus; STG, superior temporal gyrus.

### 3.3 ToM connectome correlated with personality

**FIGURE 5:**
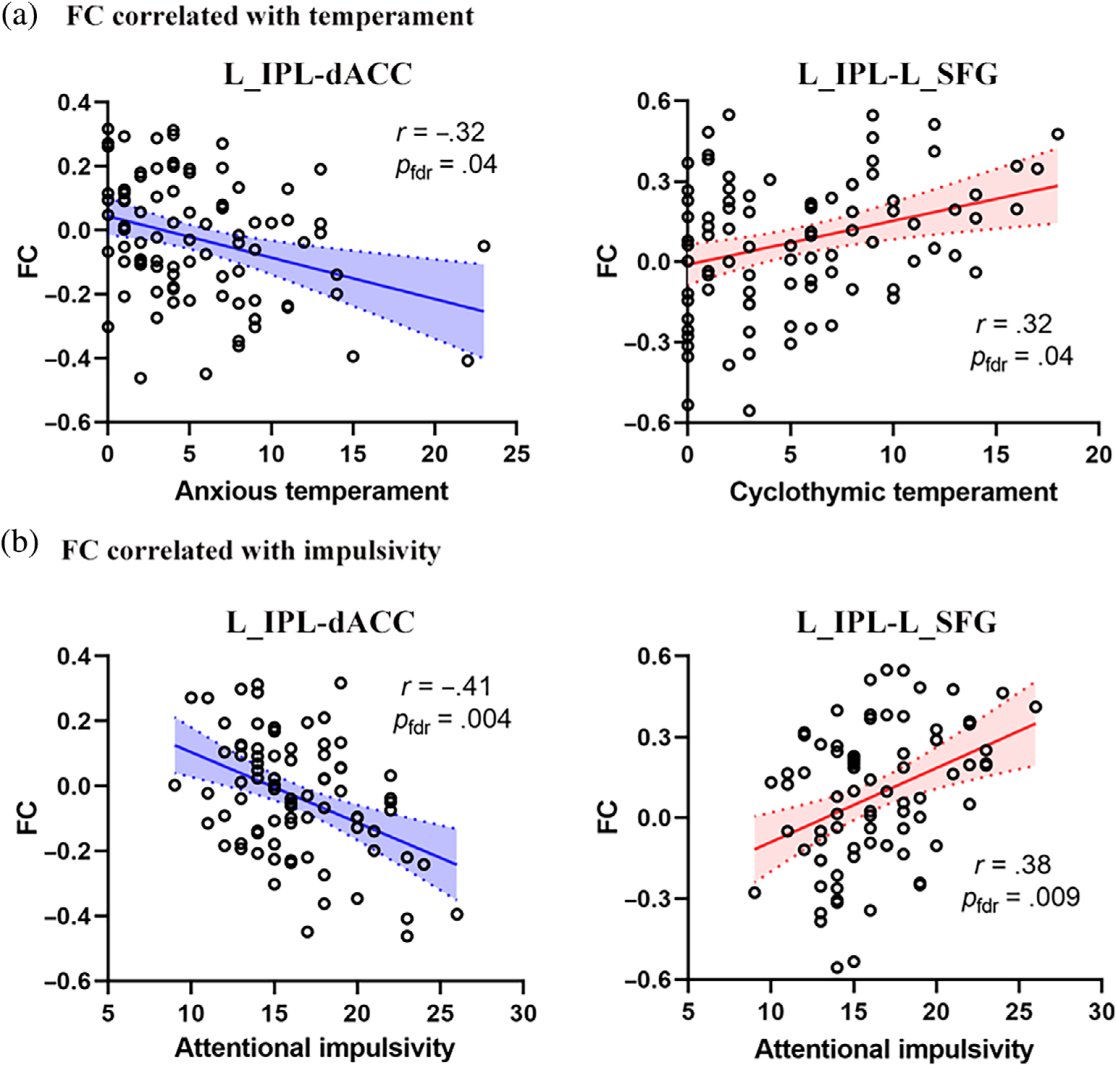
The FC correlated with temperament (a) and impulsivity (b). dACC, dorsal anterior cingulate cortex; FC, functional connectivity; IPL, inferior parietal lobule; L, left; SFG, superior frontal gyrus

The FC between left IPL and dACC was negatively correlated with anxious temperament (*r* = −.32, *p*_fdr_ = .04, Figure 5a), while the FC between left IPL and left SFG was positively correlated with cyclothymic temperament (*r* = .32, *p*_fdr_ = .04, Figure 5a). Moreover, the FC between left IPL and dACC was negatively related to attentional impulsiveness (*r* = −.41, *p*_fdr_ = .004, Figure 5b), while the FC between left IPL and left SFG was positively related to attentional impulsiveness (*r* = .38, *p*_fdr_ = .009, Figure 5b).

### 3.4 Association between CM and personality was mediated by ToM connectome

Using mediation analysis, we examined whether the relationship between CM experience and temperature (i.e., anxious and cyclothymic temperament) was mediated by the FC of IPL‐dACC and IPL‐SFG. We did not find evidence for a mediation effect.

**FIGURE 6:**
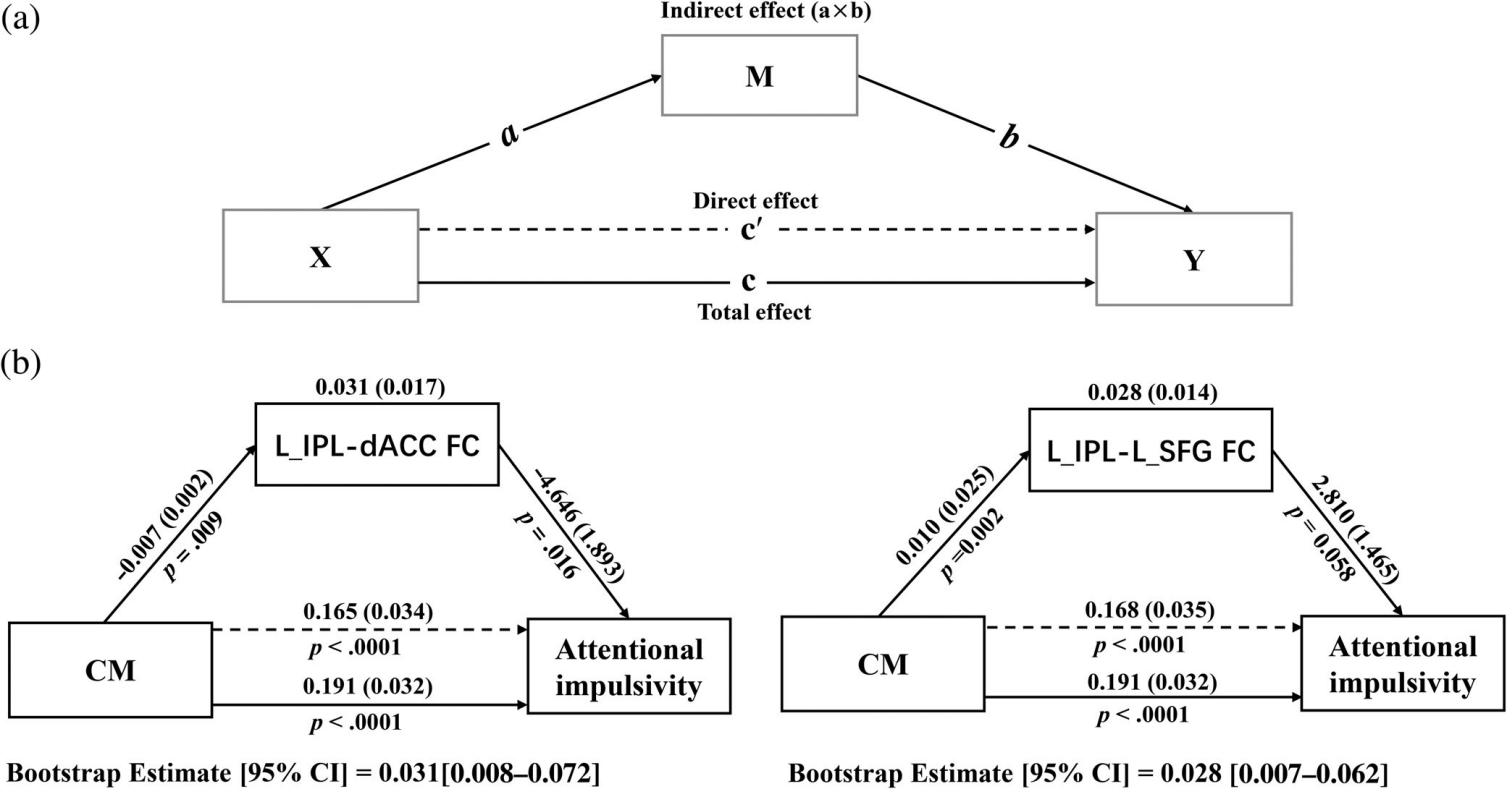
Path diagram depicting relations between CM, ToM connectome, and attentional impulsivity. (a) Standard three‐variable mediation model. Path c is the total effect of X on Y, path c′ is the direct effect of X on Y after controlling for M, and path a×b is the indirect effect of X on Y through M. (b) The FCs between IPL and dACC/SFG mediated the relationship between CM and attentional impulsivity. Path coefficients were displayed along with standard errors and the significance levels (i.e., *p* value). The 95% CI after 5,000 times bootstrap estimating the indirect effect of X on Y was listed on the bottom, suggesting the mediation was significant when not containing zero. CI, confidence interval; CM, childhood maltreatment; dACC, dorsal anterior cingulate cortex; FC, functional connectivity; IPL, inferior parietal lobule; L, left; SFG, superior frontal gyrus

We also examined whether the relationship between CM experience and attentional impulsivity was mediated by the FC of IPL‐dACC and IPL‐SFG. Figure 6b showed the significant mediation effect of these FCs on the influence of CM experience on attentional impulsivity. Specially, the CM experience had a significantly indirect effects on attentional impulsivity via the FC of IPL‐dACC (indirect effect: β=0.031, 95% CI [0.008–0.072]) and via the FC of IPL‐SFG (indirect effect: β=0.028, 95% CI [0.007–0.062]), and had a significant direct effect on attentional impulsivity (direct effect: β=0.165, 95% CI [0.098–0.233]).

## 4 DISCUSSION

This study adopted a completely data‐driven and unbiased, connectome‐wide MVPA approach to examine whole‐brain resting‐state FC differences between adults with and without CM. Our results mainly showed that (a) decreased FC between the insula and STG and between the IPL and MFG, STG, and dACC, while increased FC between the IPL and cuneus and SFG in subjects with CM experience; (b) these CM‐related FCs of the IPL with dACC and SFG were correlated with the anxious and cyclothymic temperament, and attentional impulsivity; (c) the FCs between IPL and dACC and SFG mediated the relationship between CM and attentional impulsivity. These findings provide valuable neural circuit consequences of early life adversity.

### 4.1 Altered ToM connectome related with CM

It is well known that CM is associated with impairment in social cognitive including ToM (Azar, Miller, Stevenson, & Johnson, 2017). ToM is the ability to make inferences about the intentions, beliefs, and desires of the self and others, and to recognize that someone else has a mind different from one's own (Premack & Woodruff, 1978). Initial investigations into the neural basis of ToM observed the areas (including PFC, STG, IPL, ACC, insula, precuneus, and occipital cortex) concerned with imitation, attention, complex perceptual and emotion recognition, intention understanding, and executive functions (Carrington & Bailey, 2009; Korkmaz, 2011; Wang et al., 2017). The dorsolateral PFC area plays an important role in cognitive ToM processing, such as working memory and executive control function (Abu‐Akel & Shamay‐Tsoory, 2011); the STG and IPL are essential areas in attribution (Wang et al., 2015; Zaitchik et al., 2010) and attention control (Shapiro, Hillstrom, & Husain, 2002; Wang, Zhang, et al., 2016); the insula and ACC regions mediate the understanding of other's emotions (Rizzolatti & Fabbri‐Destro, 2008); and the occipital regions integrate visual information and through reciprocal connections contributing to conscious awareness (Silvanto, Lavie, & Walsh, 2005; Wang, Tian, et al., 2016). These ToM related regions are closely associated with CM. For example, meta‐analyses reported that CM was related to altered cortical thickness and gray matter volume in the PFC, ACC, STG, insula, IPL, and occipital regions (Lim et al., 2014; Tozzi et al., 2020). Maltreatment participants showed increased activation in the IPL and occipital cortex during inhibitory control (Bruce et al., 2013), increased STG and insula activation during emotional stimuli (Blair et al., 2019; McCrory et al., 2011), and increased ACC activation during reward prediction error (Eckstrand et al., 2019). In addition, CM was also linked to decreased regional homogeneity and fractional amplitude of low frequency fluctuation in the IPL and STG (Lu, Gao, et al., 2017; Lu, Pan, et al., 2017; Philip, Kuras, et al., 2013). Overall, previous findings suggested that CM influenced the structure and function of ToM regions in adults.

Interestingly, decreased FC between insula and STG, and between IPL and STG, MFG, and dACC, and increased FC between IPL and cuneus and SFG were found in adults with CM experience. Extending previous regional findings and changed FC within ToM network only in women with CM (Fletcher & Schurer, 2017), our whole‐brain findings revealed that CM impaired the FC within ToM network not only in women but also in men. Moreover, the physical neglect specifically altered FC between PI and dAI, and emotion neglect specifically altered FC between IPL and cuneus were identified. The FC between IPL and frontal regions is implicated in attention control and decision‐making processes (Chen et al., 2013), the FC between IPL and visual regions subserves visual attentional processing (Numssen, Bzdok, & Hartwigsen, 2021), and the FC between insula and STG is involved in internal‐focused attention (Jang et al., 2018). Previous studies have found reduced FCs of the IPL with visual regions (Philip, Kuras, et al., 2013) and ACC (Eckstrand et al., 2019) were related to early life trauma exposure. Of note, CM‐related reduced FC between the IPL and dorsolateral PFC during sustained attention was moderated by SNP rs3800373 (Hart et al., 2017). In addition, network analysis reported that CM was associated with changed degree centrality in the insula, ACC, and occipital cortex (Teicher, Anderson, Ohashi, & Polcari, 2014), and decreased nodal clustering coefficient in the STG and middle occipital gyrus during behavioral inhibition (Kim, Kim, Shim, Im, & Lee, 2018). Therefore, our finding of changed ToM connectomes implied that CM experience disrupted the function of ToM network, thus resulting in maladjusted ToM abilities which are commonly seen in maltreatment persons.

Notably, the CM‐induced changed ToM connectomes strongly resembled findings in patients with psychiatric disorder (Bora & Berk, 2016; Cui et al., 2017). Especially, the FC between IPL and dACC was negatively correlated with anxious temperament, while the FC between IPL and SFG was positively correlated with cyclothymic temperament. This was understandable given that CM induced decreased FC of IPL‐dACC and increased FC of IPL‐SFG, as well as a higher level of anxious and cyclothymic temperament in our study. The present finding was consistent with a previous study that trauma‐related reduced FC between IPL and ACC was associated with heightened affective and anxiety symptoms (Eckstrand et al., 2019). As the personality traits, a higher level of anxious temperament means an increased risk of developing anxiety and depressive disorders (Kalin, 2017), whereas a higher level of cyclothymic temperament is one of the prodromal states of bipolar disorder (Syrstad et al., 2020). Accordingly, we boldly speculated that the altered cognitive ToM connectomes might be the underlying neural mechanism for how maltreatment increases the risk for the development of psychiatric disorders such as major depressive disorder, anxiety disorder, and bipolar disorder.

### 4.2 Association between CM and attentional impulsivity was mediated by ToM connectome

CM was associated with greater impulsivity, a core personality trait that is characterized by the tendency to respond to internal or external stimuli irrespective of the potential negative consequences (Moeller, Barratt, Dougherty, Schmitz, & Swann, 2001). Moreover, the FCs between IPL and dACC/SFG were correlated with individual variation in attentional impulsivity, a trait indexes instability of attention and cognitive (Netto et al., 2016). Although the cross‐sectional study withheld us from drawing any conclusions on causality, the mediation analysis revealed that the FCs between IPL and PFC/dACC mediated the relationship between CM and attentional impulsivity. This was a striking finding which could account for how CM led to the development of impulsivity. Notably, the present result verified the model proposed by Liu (2019) in a meta‐analysis. That is, early maltreatment experiences disrupted neural development, particularly in the frontoparietal system (i.e., cognitive ToM) governing response inhibition and cognitive control (Chen et al., 2013), which in turn, might result in greater attentional impulsive tendencies later in life. In fact, previous studies have documented similar findings that the white matter tract (e.g., cingulum and accumbofrontal) integrity mediated the association between child abuse and trait anxiety (Tendolkar, Martensson, Kuhn, Klumpers, & Fernandez, 2018) and negative feedback sensitivity (Kennedy et al., 2021). It suggested that CM triggered neurocognitive alterations, especially, parietal–frontal circuits alteration that heightens impulsivity and increases vulnerability to risky behavior. Our findings will hopefully encourage further research to elaborate the causality linking overall CM, as well as its specific subtypes, brain connectivity, and individual difference in personality, which may inform new formulations of the etiology of psychopathology.

## 5 LIMITATION

The present findings should be interpreted in light of some limitations. Firstly, CM experience was measured retrospectively through a self‐report questionnaire and was therefore sensitive to subjectivity and recall biases. Secondly, all participants of our study were well‐educated academics. Although the education level was included as a covariate during statistical analyses, it was still difficult to fully exclude the effect of education on our results. That is mainly due to the protective effect of education on socialization, cognitive, and brain health (Foubert‐Samier et al., 2012), and well‐education facilitates mitigate the negative impact of childhood adversity on life (Jung, Herrenkohl, Skinner, & Rousson, 2021; Zielinski & Bradshaw, 2006). Thus, the influence of CM on the brain in adults with low‐level of education should be investigated in the future. Thirdly, our study did not include task‐based fMRI data, some caution was warranted in interpreting the functional and behavioral significance of ToM network. Finally, not all maltreated children experience negative consequences of CM, some children seem like to be more resilient (Afifi & MacMillan, 2011). The cross‐sectional study made it difficult to determine whether the changed ToM connectomes were disruptive (e.g., inducing the onset of psychiatric disorder) or adaptive effect (i.e., attributing to the neuroplasticity of the brain) following CM, future longitudinal study is needed to clearly figure out this issue.

## 6 CONCLUSION

Using data‐driven whole‐brain FC method in previously maltreated but healthy adults, the present findings suggested the enduring impact that child adversity had on brain connectivity within ToM network, and offered a neuropathological model showing the relationship among CM, cognitive ToM connectome, and personality trait. These revealed the neurobiological mechanism underlying impaired ToM abilities in adults with CM, and pointed to a new framework to explain how CM leads to the development of impulsivity. Furthermore, our findings also provided new clues to the prevention and intervention strategy to reduce the risk of the development of psychopathology.

## CONFLICT OF INTEREST

The authors declare no conflicts of interest.

## Supporting information


**Appendix S1** Supporting Information.

## Data Availability

All data and code used for data analysis are available upon request.
