# Supplementary material for: Enduring effect of abuse: Childhood maltreatment links to altered theory of mind network among adults
Source: Hum Brain Mapp. 2022 Jan 28;43(7):2276–88. doi: 10.1002/hbm.25787 (PMC8996351; doi:10.1002/hbm.25787)
Supplement: Supplementary file 1 — Appendix S1 Supporting Information. [file HBM-43-2276-s001.docx]

**Enduring effect of abuse: childhood maltreatment links to altered theory of mind network among adults**

Yajing Pang, Shanshan Zhao, Zhihui Li, Nan Li, Jiarui Yu, Rui Zhang, Fengmei Lu, Heng Chen, Fengchun Wu, Wei Zheng, Jingjing Gao, Yongfeng Yang^*^, Huawang Wu^*^, Jiaojian Wang^*^

**The effect of the kind of CM exposures on FC and personality**

To examine the effect of the kind of CM exposures on the FC and personality, individuals with CM were divided into two subgroups with one group included subjects who experienced only one kind of CM exposure (i.e., Single CM), and the other group included subjects who experienced at least two kinds of CM exposures (i.e., Multiple CM). Table S1 showed that the more types of CM exposures experienced, the higher level of temperament and impulsivity subjects had. Moreover, the kind of CM exposures did not influence the FC results, as both CM subgroups showed the same group differences compared to non_CM group (Figure S1).

Table S1. Group difference of personality profile among non_CM group, single CM exposure group, and multiple CM exposures group

| Variables | Non_CM  (n = 50) | Single CM  (n = 22) | Multiple CM  (n = 18) |
| --- | --- | --- | --- |
| TEMPS-A |  |  |  |
| Total scores | 26.04 $\pm$ 9.97 | 33.86 $\pm$ 13.64 | 43.83 $\pm$ 16.93 |
| Depressive temperament | 5.86 $\pm$ 2.26 | 7.32 $\pm$ 3.47 | 10.56 $\pm$ 3.24 |
| Cyclothymic temperament | 3.60 $\pm$ 3.71 | 6.18 $\pm$ 4.58 | 9.39 $\pm$ 5.15 |
| Hyperthymic temperament | 10.94 $\pm$3.54 | 11.32 $\pm$ 4.12 | 10.11 $\pm$ 5.19 |
| Irritable temperament | 1.46 $\pm$ 1.96 | 3.50 $\pm$ 3.14 | 5.22 $\pm$ 3.90 |
| Anxious temperament | 4.18 $\pm$ 3.52 | 5.45 $\pm$ 4.33 | 8.56 $\pm$ 6.47 |
| BIS |  |  |  |
| Total scores | 54.6 $\pm$ 14.12 | 67.8 $\pm$ 10.92 | 69.88 $\pm$ 7.34 |
| Attentional impulsivity | 16.04 $\pm$ 4.99 | 17.57 $\pm$ 3.54 | 18.88 $\pm$ 3.26 |
| Motor impulsivity | 20.34 $\pm$ 4.61 | 22.62 $\pm$ 4.98 | 22.69 $\pm$ 3.26 |
| Non-planning impulsivity | 24.38 $\pm$ 4.76 | 27.37 $\pm$ 3.69 | 28.31 $\pm$ 2.85 |

Values are mean ± standard deviation.

TEMPS_A, Temperament Evaluation of Memphis, Pisa, Paris, and San Diego Auto-questionnaire; BIS, Barratt Impulsiveness Scale; CM, childhood maltreatment.


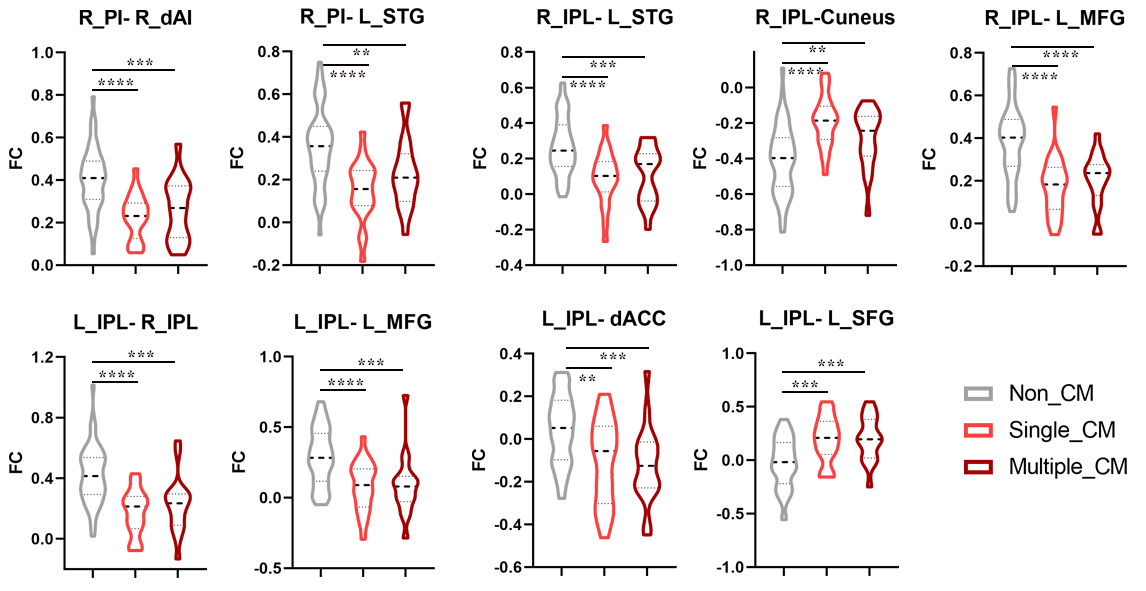


Figure S1. Group difference of FC among non_CM group, single CM exposure group, and multiple CM exposures group. *****p* < 0.0001, ****p* < 0.001, ***p* < 0.01. PI, posterior insula; dAI, dorsal anterior insula; STG, superior temporal gyrus; IPL, inferior parietal lobule; MFG, middle frontal gyrus; dACC, dorsal anterior cingulate cortex; SFG, superior frontal gyrus; CM, childhood maltreatment; R, right; L, left.

**Relationship between changed FC and the types of CM**

In order to further examine the relationship between changed FC and each type of CM, partial correlation analyses were conducted between altered FC and CM subscales in participants with CM experience, with age, gender, and years of education as covariates. The statistical significance *p* < 0.05 (uncorrected) was set. As shown in Figure S2, the FC between PI and dAI was positively correlated with physical neglect (*r* = 0.34, *p* = 0.03), while the FC between IPL and cuneus was negatively correlated with emotion neglect (*r* = -0.36, *p* = 0.02).


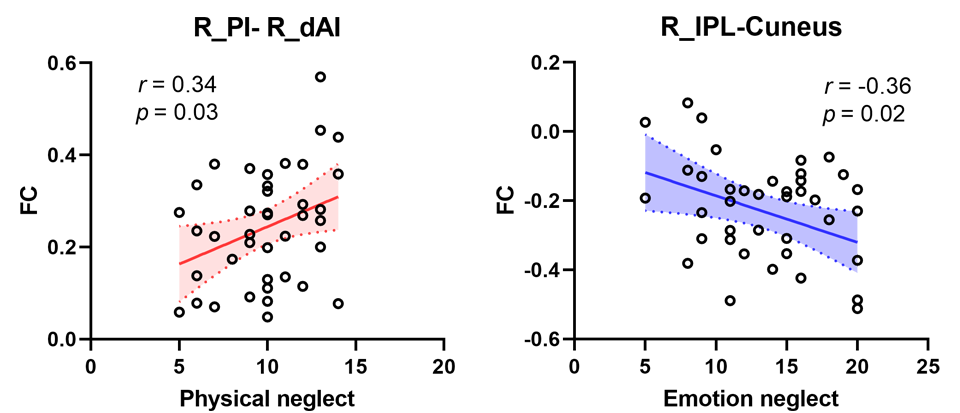


Figure S2. The relationships between changed FC and CM subscales. FC, functional connectivity; CM, childhood maltreatment; IPL, inferior parietal lobule; PI, posterior insula; dAI, dorsal anterior insula; R, right.
